# Neural Responses to Fluoxetine in Youths with Disruptive Behavior and Trauma Exposure: A Pilot Study

**DOI:** 10.1089/cap.2020.0174

**Published:** 2021-10-14

**Authors:** Soonjo Hwang, Unsun Chung, Yongmin Chang, Eunji Kim, Ji-Woo Suk, Harma Meffert, Christopher Kratochvil, Ellen Leibenluft, James Blair

**Affiliations:** ^1^ Department of Psychiatry, University of Nebraska Medical Center, Omaha, Nebraska, USA.; ^2^ Department of Psychiatry and Department of Radiology, Kyoungbook National University Hospital, Daegu, Republic of Korea.; ^3^ Target Holding, Groningen, The Netherlands.; ^4^ Emotion and Development Branch, National Institute of Mental Health, Bethesda, Maryland, USA.; ^5^ Center for Neurobehavioral Research, Boys Town National Research Hospital, Boys Town, Nebraska, USA.

**Keywords:** disruptive behavior disorder, trauma, fluoxetine, insula, ventromedial prefrontal cortex

## Abstract

***Objective:*** A preliminary investigation of the impact of a serotonergic agent (fluoxetine) on symptom profile and neural response in youths with disruptive behavior disorders (DBDs) and a history of trauma exposure.

***Methods:*** There were three participant groups: (i) Youths with DBDs and trauma exposure who received fluoxetine treatment for 8 weeks (*n* = 11); (ii) A matched group of youths with DBDs and trauma exposure who received routine regular follow-up in an outpatient clinic (*n* = 10); and (iii) Typically developing youths (*n* = 18). All participants conducted an expression processing functional magnetic resonance imaging task twice, 8 weeks apart: (pretreatment and post-treatment for youths with DBDs).

***Results:*** Youths with DBDs and trauma exposure who received fluoxetine treatment compared to the other two groups showed: (i) significant improvement in externalizing, oppositional defiant disorder, irritability, anxiety-depression, and trauma-related symptoms; (ii) as a function of fearful expression intensity, significantly decreased amygdala response and increased recruitment of regions implicated in top-down attention control (insula cortex, inferior parietal lobule, and postcentral gyrus) and emotional regulation (ventromedial prefrontal cortex [vmPFC]); and (iii) correlation between DBD/irritability symptom improvement and increased activation of top-down attention control areas (inferior parietal lobule, insula cortex, and postcentral gyrus) and an emotion regulation area (vmPFC).

***Conclusions:*** This study provides preliminary evidence that a serotonergic agent (fluoxetine) can reduce disruptive behavior and mood symptoms in youths with DBDs and trauma exposure and that this may be mediated by enhanced activation of top-down attention control and emotion regulation areas (inferior parietal lobule, insula cortex, and vmPFC).

## Introduction

Childhood trauma and maltreatment are the most common forms of early life stress (ELS) and are associated with significant mental health risks (Agorastos et al. 2019). Large epidemiological studies report that more than one in four children experience a significant traumatic event such as child abuse, domestic/community/school violence, vehicular accident, or other shocking/terrifying experiences (Costello et al. 2002; Cohen et al. 2010).

These children are at increased risk for the development of significant and potentially long-lasting mental health problems (Cohen et al. 2010), including internalizing (anxiety, depression) and, of particular interest here, externalizing (aggression) behavior (Thornberry et al. 2001; Lansford et al. 2002). Indeed, a significant proportion (up to 15%–20%) (Bernhard et al. 2018) of children/adolescents with conduct disorder/oppositional defiant disorder (CD/ODD; the childhood diagnoses associated with externalizing behavior) have a history of trauma exposure (Steiner et al. 2011).

Current treatment options for youths with CD/ODD are limited (Cohen et al. 2010), and minimal data have explored how treatment impacts neural areas implicated in the pathophysiology. The current preliminary study aims to address this gap by investigating the impact of a serotonergic agent (fluoxetine) on symptom profile change and neural response in youths with disruptive behavior disorders (DBDs) and a history of trauma exposure.

There are suggestions that serotonergic function is disturbed in youths with DBDs (Chang et al. 2017). For example, it has been reported that lower serotonergic responsivity in children with attention-deficit/hyperactivity disorder may predict the development of CD in the future (Chang et al. 2017). More broadly, considerable data from both preclinical and human studies indicate that disrupted serotonergic function might be associated with an increased propensity for aggression, particularly anger-based reactive aggression (Miczek et al. 2002; Flory et al. 2007; Coccaro 2012; Chang et al. 2017). Preliminary data indicate that acute and chronic administration of the serotonergic agent (i.e., trazodone) may reduce aggression and impulsivity in children with DBDs (Zubieta and Alessi 1992). Similarly, another Selective Serotonin Reuptake Inhibitor (SSRI) citalopram, added to methylphenidate, reduced chronic severe irritability in youths more than did methylphenidate alone (Towbin et al. 2020).

There are also indications that exposure to ELS disrupts serotonergic function (Puglisi-Allegra and Andolina 2015; Houwing et al. 2017). Specifically, there is a long-standing, although debated, literature suggesting that exposure to ELS interacts with specific serotonergic polymorphisms to increase the risk for aggression (Houwing et al. 2017). In addition, posttraumatic stress disorder (PTSD), an anxiety condition that emerges subsequent to maltreatment and stress exposure, has also been associated with serotonergic dysfunction (Maes et al. 1999; Houwing et al. 2017). Treatment studies have shown that serotonergic agents can reduce PTSD symptoms in children (Cohen et al. 2010; Huemer et al. 2010). In short, the literatures on both the treatment of DBDs and the impact of ELS suggest that a serotonergic agent (in this study, fluoxetine) might be beneficial for reducing symptomatology in youths with DBDs who also have a history of trauma exposure.

In this regard, an area that has received almost no empirical attention is how treatment of DBDs impacts cognitive and affective functions at the neural level; that is, there is a minimal understanding of the mechanistic change in neural areas by which successful intervention is achieved. Yet, such data are critical to specify precisely the pathophysiology of these disorders as treatment targets (target engagement). In this regard, there are two forms of neurocognitive dysfunctions commonly seen in youths with DBDs: First, an increased responsiveness to negative emotional stimuli/acute threat (this is particularly related to an increased risk for anger-based reactive aggression) (Viding et al. 2012; Hwang et al. 2016). Pertaining to our study aim, such increased responsiveness is also seen as a consequence of exposure to ELS (VanTieghem and Tottenham 2018; Santiago et al. 2018). It may reflect increased amygdala sensitivity to negative emotional stimuli/threat cues and/or dysfunction in ventromedial prefrontal cortex's (vmPFC) putative role in inhibiting the amygdala's response (for an extensive review, Andrews and Jenkins 2019).

Previous studies in children exposed to ELS consistently report increased amygdala responses to threat (Di Iorio et al. 2017; Kaiser et al. 2018) and decreased activation of vmPFC to emotional stimuli (Blair 2013). The second form of neurocognitive dysfunction commonly seen in youths with DBDs involves impaired recruitment of regions implicated in top-down attention control and emotion regulation (Leibenluft 2017; Blair 2018) dorsolateral prefrontal, parietal, and insula cortices (Buhle et al. 2014). This form of dysfunction is also seen as consequence of exposure to ELS (Blair 2013; Blair et al. 2019).

Notably, brain regions such as the amygdala, ventromedial frontal cortex, and dorsolateral prefrontal, parietal, and insula cortices show high concentrations of serotonin receptors (Schotte et al. 1983; Ishii et al. 2015; da Cunha-Bang et al. 2018). The SSRI fluoxetine might plausibly augment the functions of these neural areas and, if their dysfunction is causally related to DBD symptoms, thus be associated with symptom improvement. The current study provides pilot data on this issue in the context of the child and adolescent psychiatry outpatient setting of a tertiary care hospital. Youths with DBDs and a history of trauma exposure received standard psychiatric care that either included or did not include fluoxetine. Symptom assessment and blood oxygen level dependent (BOLD) responses to fearful expression stimuli were recorded pre- and post 8 weeks of treatment. A comparison group of healthy youth, who were also scanned twice 8 weeks apart, allowed an index of test–retest effects.

We predicted that, following fluoxetine treatment, youths with DBDs and ELS exposure would show: (i) significant improvement in their DBD symptoms (externalizing problems, ODD symptoms, CD symptoms, irritability, aggression, and breach of rules measured by Child Behavior Checklist [CBCL]) (Achenbach 1991), as well as trauma-related symptoms (measured by Child Report of Post-Traumatic Symptoms [CROPS] and Parent Report of Post-Traumatic Symptoms [PROPS]) (Strand et al. 2005); (ii) improvement in dysfunctional responding within neural areas implicated in emotional responding and top-down attention control; and (iii) correlation between improvement in dysfunctional neural responding and the degree of symptom improvement.

## Methods

### Participants

Fifty-one participants, aged 10–18, were recruited from a university hospital child and adolescent psychiatry outpatient clinic (*n* = 32) and the surrounding local community (*n* = 19). Participants were divided into two groups: (i) Patients with DBDs (ODD or CD) and a history of significant trauma exposure and (ii) healthy comparison youths. The Institutional Review Board of Kyoungbook National University Hospital approved the study. For clinical assessment and characterization, see Supplementary Material Section S1.

### Symptom profile measurement

Symptom profiles were assessed at the initial assessment session and the final assessment session after 8 weeks of study participation. Parents completed the CBCL for internalizing/externalizing problems (Achenbach 1991). Posttraumatic Symptoms were measured by CROPS and PROPS (Strand et al. 2005). For these scales, the Korean-translated versions have been standardized with adequate psychometric properties that are consistent with those reported in U.S. children/adolescents (Oh and Lee 1990; Lee et al. 2011). For exclusion criteria, see Supplementary Material Section S2.

### Medication treatment (fluoxetine)

This was an open-label clinical trial of fluoxetine for youths with DBDs and history of trauma exposure, combined with functional magnetic resonance imaging (fMRI) scans before and after treatment. After their initial assessment by the child and adolescent psychiatrist (Dr. U.C.), participants' families were invited to join the study, if the treating/assessing clinician determined that the youth showed significant levels of symptoms and a history of trauma exposure. Treatment with fluoxetine was then discussed with the family. Families choosing fluoxetine (*n* = 11) received, for 8 weeks, this treatment combined with routine standard psychiatric care. Families not choosing fluoxetine (*n* = 10) received routine standard psychiatric care for 8 weeks. Routine standard psychiatric care included weekly appointments at the outpatient child and adolescent psychiatry clinic. These appointments involved psychiatric symptom assessments and supportive therapy (discussions of recognition of psychiatric symptoms, development of coping skills, and enhancing daily functioning); see Supplementary Material Section S3 for further details. None of the youths with DBDs in the routine standard psychiatric care was taking any psychiatric medications, including fluoxetine.

MRI sessions took place after the initial assessment session but before initiation of fluoxetine treatment (for the participants scheduled for fluoxetine treatment) and after the final postintervention assessment session (8 weeks later). To note, the research personnel who performed the MRI procedure were blinded of the treatment status of the participants. For the youths with DBDs, treatment continued at the outpatient clinic after their completion of study participation. All of the youths with DBD remained in the clinic for follow-up treatment after study completion.

### Experimental design

**FIG. 1.**
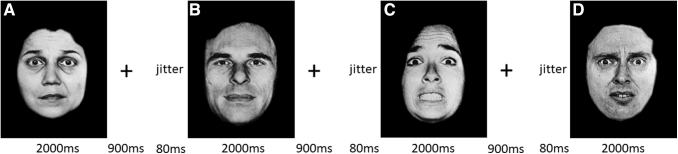
Facial expression task. In this task, all the participants were asked to determine the gender of the faces. Faces are presented with parametrically modulated intensity [25% **(A)**, neutral **(B)**, 150% **(C)**, and 100% **(D)**] of fearful expressions.

The fearful facial expression task was adapted from previous work (Fig. 1) (Marsh et al. 2008). Specifically, participants were shown photographs of faces of 10 men and women from the Pictures of Facial Affect series (Tottenham et al. 2009) displaying either neutral expressions or fearful facial expressions of differing intensities (50%, 100%, or 150%). The levels of fear intensity were achieved by morphing neutral expressions into fearful expressions to create composites (50% or 100% intensity) or by extrapolating from the fearful expressions to create exaggerated expressions (150% intensity).

Each trial started with the presentation of a face for 2000 ms, after which a fixation cross (interstimuli interval) was displayed for 900 ms. Participants were asked to identify the sex of the face by button press. Each run consisted of 80 face trials, which were randomly interspersed with 80 fixation trials (900 ms) to jitter stimulus presentation. The total task involved one run which lasted 5 minutes and 50 seconds. For further details, see Supplementary Material Section S4.

### Image acquisition

Participants were scanned using a 3.0-Tesla GE discovery 750w MRI scanner (GE Healthcare, Milwaukee, WI). Following sagittal localization, functional T2* weighted images were acquired using an echo-planar single-shot gradient echo pulse sequence (repetition time [TR] = 2500 ms, echo time [TE] = 27 ms, flip angle 90°, field-of-view [FOV] = 240 mm, 94 × 94 matrix, 2.6 × 2.6 × 2.5 mm voxels). Images were acquired in 43 slices of 2.5 mm per brain volume (distance factor 21%), with each run lasting 5 minutes 50 seconds. In the same session, a high-resolution T1-weighted anatomical image was acquired to aid with spatial normalization (TR = 2200 ms, TE = 2.48 ms, flip angle = 8°, FOV = 230 mm, 0.9 × 0.9 × 0.1 mm^3^ voxels, 176 axial slices, 256 × 208 matrix, thickness = 1.0 mm, distance factor 50%) in register with the echo-planar imaging data set was obtained covering the whole brain. For image data preprocessing, Supplementary Material Section S5.

### Statistical analysis

#### Clinical characteristics

Two multivariate analyses of variance (MANOVAs) were conducted on the symptom profiles. The first examined group differences in the nine symptom types of interest (externalizing problems, breach of rules, aggressive behavior, ODD symptoms, CD symptoms, irritability, anxiety depression, and trauma-related symptoms measured by CROPS and PROPS) at baseline. The second examined group differences in symptom change post-treatment relative to pretreatment.

#### Behavioral data

We conducted a full three (group: healthy, youths with DBD and fluoxetine treatment, and youths with DBD and without fluoxetine treatment) by two (time: initial and follow-up) repeated analysis of covariance on the accuracy and reaction time data with intelligence quotient (IQ), sex, and age as covariates.

#### MRI data

We selected two approaches to the data analyses of BOLD responses: First, we focused on the amygdala and vmPFC. As noted, previous work has indicated atypical amygdala and vmPFC responding to negative emotional stimuli in patients with DBDs (Viding et al. 2012; Hwang et al. 2016) and as a consequence of exposure to ELS (VanTieghem and Tottenham 2018; Santiago et al. 2018). An initial *t*-test on the baseline data was conducted to identify whether amygdala and vmPFC regions of interest (ROIs) could be identified that differentiated neural responses between youth with DBDs pretreatment and healthy comparison adolescents at baseline. Treatment effects were then examined in the identified ROIs through two (group: youths with DBDs and fluoxetine treatment and youths with DBDs and without fluoxetine treatment) by two (time: initial and follow-up) analysis of variances (ANOVAs) within Statistical Package for the Social Sciences (version 26) on the facial expression weighted BOLD responses. Note that because any ROIs identified were generated from a DBD versus healthy contrast in baseline, they would be focused on the roles of vmPFC and amygdala in the pathology of DBD/ELS (Andrewes and Jenkins 2019) but unbiased with respect to pre/post-treatment differences in the groups with DBDs.

Second, we conducted a three (groups: youths with DBDs and fluoxetine treatment, youths with DBDs and without fluoxetine treatment, healthy comparison adolescents) by two (times: initial and follow-up) ANOVA with covariates (i.e., age, sex, and IQ) on the whole brain, facial expression weighted BOLD response data through 3dMVM. Correction for multiple comparisons was performed using a spatial clustering operation in 3dClustSim embedded in analysis of functional neuroImages utilizing the auto-correction function (-acf) with 10,000 Monte Carlo simulations for a whole brain gray matter mask. The initial threshold was set at *p* = 0.001 (Cox et al. 2017). This procedure yielded an extant threshold of *k* = 23 voxels, which then resulted in a cluster-level false-positive probability of *p* < 0.05, corrected for multiple comparisons. To facilitate future meta-analytic work, effect sizes (Partial *η*^2^) for all clusters are reported.

#### Relationship of treatment-related BOLD response changes to symptom level changes

These were examined using correlational analyses; differential BOLD responses to intensity-weighted fearful expressions (post- minus pretreatment) were correlated against symptom level changes (i.e., post- minus pretreatment).

## Results

### Clinical characteristics

Two youths with DBDs were excluded from the study due to IQ lower than 70 (one from the group with, and one from the group without, fluoxetine treatment). Five youths in the DBDs without fluoxetine treatment group were withdrawn from the study (one required inpatient hospitalization in week 5, one developed otitis media in week 3, and three decided to initiate a medication treatment in weeks 2–5). Four youths with DBDs and one healthy youth were not included in the final analyses, due to excessive movement during the scanning. Thus, we included 18 healthy youths, 11 youths with DBDs who received fluoxetine treatment, and 10 youths with DBD without fluoxetine treatment in the final analyses. For the demographic characteristics, see Table 1.

**Table 1.** Demographics and Characteristics

	Healthy youths (*n* = 18) Mean (SD)	Youths with disruptive behavior disorders on fluoxetine treatment (*n* = 11)	Youths with disruptive behavior disorders without fluoxetine treatment (*n* = 10)	*p*-Value
Age	15.2 (1.5)	14.8 (0.9)	15.7 (0.7)	0.26
Sex	5/13 (male/female)	6/5	6/4	0.18
IQ	101.9 (7.0)	97.5 (7.8)	96.1 (6.0)	0.08
Diagnosis				
ADHD		7 (63.6%)	6 (60%)	
ODD		9 (81.8%)	7 (70%)	
CD		4 (36.4%)	3 (30%)	
MDD		3 (27.3%)	3 (30%)	
AD NOS		2 (18.2%)	3 (30%)	
PTSD		1 (9.0%)	2 (20%)	
Fluoxetine Average dose		26 mg (8.2)		

ADHD, attention-deficit/hyperactivity disorder; AD NOS, anxiety disorder, not otherwise specified; CD, conduct disorder; IQ, intelligence quotient; MDD, major depressive disorder; ODD, oppositional defiant disorder; PTSD, posttraumatic stress disorder; SD, standard deviation.

### Symptom profiles in baseline and symptom profile changes in follow-up

Unsurprisingly, our first MANOVA on group differences in symptoms at baseline was significant (*F* = 4.305, *p* < 0.001; *η*^2^ = 0.572) with significant group differences for all nine symptom types [*F*(2,36) = 8.019–159.593, *p* = 0.001 to <0.001; *η*^2^ = 0.308–0.899]; for full details, see Supplementary Table S1. Follow-up contrasts (*t*-tests) revealed that for all nine symptom types, both groups of youths with DBDs (those who would take fluoxetine and those who would not) showed significantly higher levels of symptom severity compared to the healthy comparison participants (mean differences: 4.57–32.29; *p* = 0.018 to <0.001). Notably, though, the groups of youths with DBDs (those who would take fluoxetine and those who would not) did not significantly differ for any symptom type (mean differences: 0.046–5.15; *p* = 0.324–0.999); for full details, see Supplementary Table S1.

Importantly, our second MANOVA conducted on symptom change (post- vs. pretreatment) was also significant (*F* = 3.100, *p* = 0.001; *η*^2^ = 0.490) with group differences for all symptoms [*F*(2,36) = 3.442–54.104, *p* = 0.043 to <0.001; *η*^2^ = 0.161–0.750] except breach of rules and CD symptoms [*F*(2,36) = 0.714 and 1.485, *p* = 0.497 and 0.240, *η*^2^ = 0.038 and 0.076, respectively]; for full details, see Supplementary Table S2. Follow-up contrasts revealed that for all significant symptom types, youths with DBDs treated with fluoxetine showed greater symptom reductions than both the youth with DBDs not treated with fluoxetine (mean differences: 2.464–15.682; *p* = 0.039 to <0.001) and the healthy comparison youths (mean differences: 2.419–17.460; *p* = 0.033 to <0.001). In contrast, in youth with DBDs not treated with fluoxetine and the healthy comparison participants, symptom measures did not change from baseline to follow-up (mean differences: 0.044–2.211; *p* = 0.992–0.999); for full details, see Supplementary Table S2 and Supplementary Figure S1.

### Behavioral data

Behavioral data indicated that participants in all three groups successfully performed the identification of sex task (mean accuracy 88.1% [standard deviation, SD = 16.7] and mean reaction time = 880.64 ms [SD = 101.40]). The ANOVAs conducted on these data revealed no significant effects of group [*F*(2,35) = 1.977 and 0.028; *p* = 0.154 and 0.972; *η*^2^ = 0.102 and 0.002] or group-by-time interactions [*F*(2,35) = 1.733 and 0.016; *p* = 0.192 and 0.984; *η*^2^ = 0.090 and 0.001] for accuracy and response time (RT), respectively. There was no main effect of time for accuracy [*F*(1,35) = 0.098; *p* = 0.756; *η*^2^ = 0.003]. However, there was a main effect of time for RT [*F*(1,35) = 5.833; *p* = 0.021; *η*^2^ = 0.143]; participants were faster post- relative to pretreatment (pre: 924.83 [standard error, SE = 23.57]; post: 860.78 [SE = 17.51]).

### MRI data

#### ROI analyses

Our initial analyses were focused on the vmPFC and amygdala ROIs, which showed pretreatment differences in activity in response to facial expression intensity between youths with DBDs/history of trauma exposure and healthy comparison youths. Within these regions at baseline, the youths with DBDs (irrespective of which treatment group they would enter) showed increased amygdala responses and decreased vmPFC responses relative to healthy comparison youths [*t*(37) = 3.825 and −3.993, *p* < 0.001 *η*^2^ = 0.283 and 0.301 for the amygdala and vmPFC, respectively]; for full details, see Supplementary Material Section 6.

**FIG. 2.**
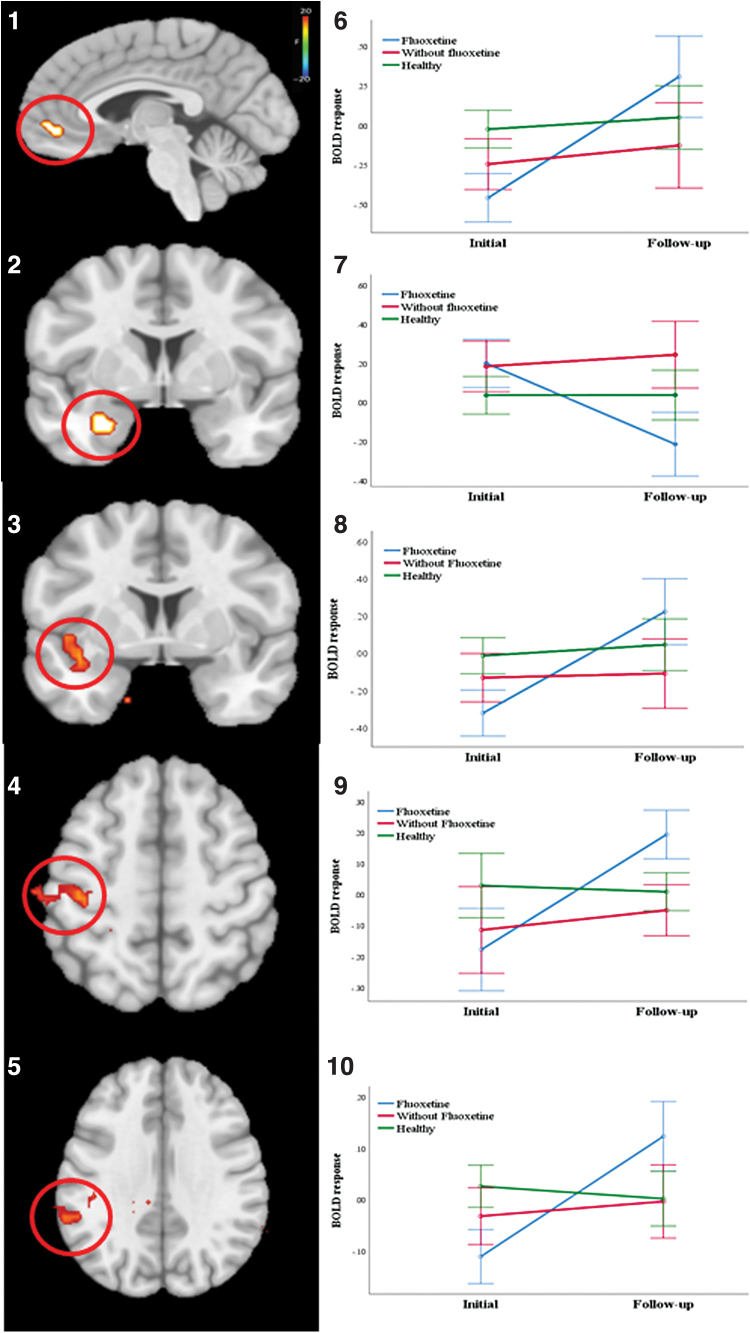
**(1)** Region of interest ventromedial prefrontal cortex (coordinates: −4, 40, −3); **(2)** ROI amygdala (coordinates: −25, −1, −21); **(3)** insula cortex (coordinates: −34, 2, −10); **(4)** postcentral gyrus (coordinates: −37, −25, 47); and **(5)** inferior parietal lobule (coordinates: −61, −34, 32) showing a significant group by time interaction; **(6–10)** In all those areas except amygdala, weighted BOLD responses to fearful expression were significantly increased in youths with DBDs who received fluoxetine after the treatment, compared to youths with DBDs and without fluoxetine treatment, as well as healthy youths. In amygdala, weighted BOLD responses to fearful expression were significantly decreased in youths with DBDs who received fluoxetine after the treatment, compared to youths with DBDs and without fluoxetine treatment [BOLD responses: **(6)** ventromedial prefrontal cortex, **(7)** amygdala, **(8)** insula cortex, **(9)** postcentral gyrus, and **(10)** inferior parietal lobule]. BOLD, blood oxygen level dependent; DBD, disruptive behavior disorder. Color images are available online.

Notably, Group (youths with DBDs and fluoxetine treatment vs. youths with DBDs and without fluoxetine treatment) by Time (Pre- vs. post-treatment) revealed significant Group × Time interactions in both regions [*F*(1,19) = 14.516 and 6.134, *p* < 0.001 and 0.023, *η*^2^ = 0.433 and 0.244 for amygdala and vmPFC, respectively]. Youths with DBDs and fluoxetine treatment showed a greater decrease in amygdala responsiveness and a greater increase in vmPFC responsiveness as a function of facial expression intensity relative to youths with DBDs and without fluoxetine treatment; see Figure 2.

#### Whole brain analysis

Three regions showed significant Time-by-Treatment interactions: left superior temporal/insula cortex, inferior parietal lobule, and left postcentral gyrus; see Table 2. Notably, proximal regions to left superior temporal/insula cortex, inferior parietal lobule, and left postcentral gyrus all showed atypical responses in all the youth with DBD relative to the healthy comparison youth by the main effect of group (Supplementary Table S4). Within all these regions, the group of youths treated with fluoxetine showed a greater increase (post- relative to pretreatment) in their modulated BOLD response as a function of facial expression intensity than the group of participants not treated with fluoxetine (Bonferroni corrected *p* = 0.006 to <0.001) and the healthy comparison individuals (Bonferroni corrected *p* < 0.001 for all regions); see Figure 2.

**Table 2.** Brain Regions Showing Significant Interactions

Region^a^	Coordinates of peak activation^b^							
	Left/Right	BA	*x*	*y*	*z*	*F*	Voxels	η^2^
Group by time								
Superior temporal gyrus/insula cortex	Left	38/13	−34	2	−10	12.26	31	0.405
Inferior parietal lobule	Left	40	−61	−34	32	11.12	46	0.423
Postcentral gyrus	Left	3	−37	−25	47	9.97	34	0.357
ROI ventromedial prefrontal cortex^*^	Left	24	−4	40	−3	4.79	25	0.433
ROI amygdala^^**^^	Left		−25	−1	−21	3.61	10	0.244

^a^ According to the Talairach Daemon Atlas.

^b^ Based on the Tournoux and Talairach standard brain template.

^*^ *p* = 0.005; ^**^p = 0.002.

BA, Brodmann area; ROI, region of interest.

#### Correlation between BOLD response changes and symptom improvement

We examined the correlation between BOLD response changes (post- vs. pretreatment) and symptom (post- vs. pretreatment) improvement in youths with DBDs using the symptom profiles and the signal in the neural areas exhibiting significant group by time interactions (Table 3). These revealed consistent significant associations between BOLD response changes in all five regions and improvement in externalizing, aggression, ODD symptoms, and irritability (*ρ* = 0.415–0.705, *p* < 0.001–0.026) (for full details, see Table 3).

**Table 3.** Correlation Between Symptom Profile Changes (Differences Between Pre- and Post-Treatment) and Blood Oxygen Level Dependent Response Changes (Differences Between Initial Scan Pretreatment and Post-Treatment Scan)

	Externalizing problem	Aggressive behavior	CBCL ODD symptoms	CBCL irritability	CBCL anxiety and depression	CROPS	PROPS
Inferior parietal lobule	0.412	0.433^*^	0.705^**^	0.548^*^	0.17	0.215	0.143
Insula cortex	0.449^*^	0.302	0.580^**^	0.143	0.201	0.088	0.066
Posterior-central gyrus	0.379	0.227	0.509^**^	0.323	0.161	0.247	0.076
vmPFC	0.419	0.450	0.614^**^	0.456^**^	0.228	0.292	0.211
Amygdala	0.505^**^	0.415^**^	0.541^**^	0.565^**^	0.307	0.434^**^	0.197

^*^ *p* < 0.05; ^**^p < 0.01.

CBCL, child behavior checklist; CROPS, child report of posttraumatic symptoms; ODD, oppositional defiant disorder; PROPS, parent report of posttraumatic symptoms; vmPFC, ventromedial prefrontal cortex.

## Discussion

In this exploratory study, our aim was to examine the extent to which a serotonergic agent (fluoxetine) reduced symptoms associated with DBD and trauma exposure and influenced neural responding in patients with DBD and trauma exposure and the extent to which change in neural response was associated with symptom change. There were three main findings. First, administration of fluoxetine was associated with significant reductions in symptomatology for all symptom measures except rule breaches and CD symptoms. Second, youths with DBDs and history of trauma exposure who received fluoxetine treatment, compared to those who did not, showed decreased amygdala response and increased vmPFC response to fearful expression following treatment. In addition, youths who received fluoxetine treatment also showed increased activation in the insula cortex, inferior parietal lobule, and postcentral gyrus. Third, there were significant correlations between improvement in externalizing, aggression, ODD symptoms, and irritability and BOLD response changes in all five regions listed above.

Fluoxetine treatment yielded significant reductions in symptom severities. These were shown for anxiety and depression symptoms, trauma-related symptoms, and DBD symptoms. SSRIs are used as first-line treatment for anxiety and depression symptoms with considerable data attesting to their efficacy in pediatric populations (Birmaher et al. 2007). Therefore, our finding that fluoxetine decreased anxiety-depression symptoms was highly anticipated. The current finding is also in line with the previous studies showing efficacy of serotonergic agents in reducing trauma-related/PTSD symptoms in pediatric populations (Cohen et al. 2010; Huemer et al. 2010).

However, in addition, it is noteworthy that there were significant reductions in disruptive behavior symptoms (externalizing problems, aggressive behavior, and ODD symptoms) and irritability. Previous works have suggested that SSRIs are effective treatments for externalizing psychopathologies, especially reactive aggression, impulsivity (Zubieta and Alessi 1992), and irritability (Towbin et al. 2020). The current work supports and extends these previous works by potentially suggesting the effectiveness of a serotonergic agent for treatment of youths with DBDs and a history of trauma exposure. It is striking how few pharmacological studies have been conducted for youths with psychopathology related to trauma exposure, even in cases with a confirmed diagnosis of PTSD (Cohen et al. 2010). We hope the current exploratory study begins to address this sparsity.

We did not observe any significant symptom improvement in breach of rules and other CD symptoms after fluoxetine treatment. This might reflect a type II error due to the sample size but it is worth also noting that CD symptoms can develop through separable neurodevelopmental pathways and reflect different underlying psychopathologies (Blair 2013; Hwang et al. 2016). One route is additionally expressed through callous-unemotional traits (reduced guilt and empathy) (Frick 2012), a concept incorporated into the new Diagnostic and Statistical Manual (DSM-5, Diagnostic and Statistical Manual of Mental Disorders, 5th edition; American Psychiatric Association 2013) as the “limited prosocial emotions specifier.” Callous-unemotional traits are significant contributors to the exacerbation of CD symptoms (Frick 2012). It is possible that fluoxetine treatment may provide less benefit for youths with CD and with callous-unemotional traits and that this is reflected in the absence of change for CD symptoms (see also Balia et al. 2018). This will be worth formally examining in future work.

With respect to our neural findings, we observed that fluoxetine appeared to redress core aspects of the psychopathology associated with DBD and trauma exposure; that is, the increase in amygdala and decrease in vmPFC responsiveness to emotional stimuli (Blair 2013; Andrewes and Jenkins 2019). We observed these expected neural aberrations at baseline (Supplementary Table S3) when comparing the participants with DBDs and trauma exposure (irrespective of what treatment they would receive) and the healthy comparison youths. In the follow-up scans these neural aberrations were significantly impacted only in those patients who went on to receive fluoxetine. Previous work has indicated that SSRI treatment reduces hyperactivation within the amygdala (Chau et al. 2017). As such, the current data are consistent with this previous work. The current data offer encouragement that fluoxetine may ameliorate this potential core pathophysiology in youths with DBDs and trauma exposure (target engagement).

Fluoxetine also significantly increased modulation of BOLD responses by facial expression intensity within regions implicated in top-down attention control (inferior parietal lobule) and response inhibition (postcentral gyrus and insula cortex). There have been suggestions that regions implicated in top-down attention control might be dysfunctional in at least some individuals with DBDs (Leibenluft 2017; Blair 2018), and this form of dysfunction has also been reported as a consequence of exposure to ELS (Blair 2013; Blair et al. 2019). Indeed, in baseline, these regions showed decreased activation in the youths with DBDs as a group pretreatment (i.e., irrespective of future treatment) relative to the healthy comparison youths (Table S3).

It is worthwhile to notice that previous works have also indicated that SSRI treatment increases responses to emotional stimuli in regions implicated in top-down attention control, such as ventrolateral prefrontal cortex in treatment of generalized anxiety disorder with SSRI (Maslowsky et al. 2010; MacNamara et al. 2016). Indeed, one relatively recent study reported increased activation of a proximal area (temporoparietal junction) to our finding (superior temporal gyrus/insula) in response to negative emotional stimuli after SSRI treatment in adults with intermittent explosive disorder (Cremers et al. 2016). Patients with intermittent explosive disorder share a similar clinical presentation of reactive aggression and irritability to our study population (American Psychiatric Association 2013).

Finally, there were significant associations between symptom profile improvement (CBCL DBD symptoms and irritability) and treatment-related neural response changes in all regions showing significant group-by-time interactions. It should be noted that all five regions showed significant levels of correlation with CBCL ODD symptom changes that have extremely low probability (3.12 × 10^−7^). Thus, this implies reflection of genuine correlation between symptom profile changes (improvement) and neural changes observed in these areas. In short, we were able to demonstrate actual target engagement (changes in the neural areas) that is correlated with symptom profile improvements in this study group (Gorka et al. 2019). These data are important both in understanding the impact on the brain of fluoxetine in these patients but also for understanding the pathophysiology of the underlying condition. The current data perhaps indicate the particular importance of dysfunction within amygdala and its improvement correlated with the improvement of DBD/irritability symptoms. Amygdala and its dysfunction have been implicated in previous theoretical work on irritability/reactive aggression/ODD (Blair et al. 2014).

In addition, the findings of this study may potentially suggest functional changes at a network level, given that the neural areas showing significant changes by treatment are indeed parts of various networks such as default mode network (vmPFC and inferior parietal cortex), or salience network (amygdala) (Lv et al. 2018). However, we were not able to observe network level changes *per se* (e.g., amygdala finding was not accompanied with changes in the other major areas of salience network, such as thalamus or ventral striatum) most likely because we did not implement resting-state fMRI method and also with small number of participants. Future study would be warranted in this regard.

While provocative, this was a preliminary study with notable limitations. First, the sample size in each treatment arm was small (11 fluoxetine treatment, 10 regular follow-up without fluoxetine treatment, and 18 healthy youths). This may have contributed to the absence of a treatment effect of Fluoxetine on breach of rules or CD-related symptoms, for example.

Second, this was not a randomized clinical trial with blinding. However, while there might have been confounding factors in the decision-making process determining which patients received fluoxetine (including personal, cultural, and family-dynamic factors), there was no significant difference in baseline DBD symptoms between youths who received fluoxetine treatment and youths who did not. In addition, the participants, their parents, and their clinicians were aware of treatment status. However, the neuroimaging analysis team was not. As such, concerns with respect to the results of fluoxetine's treatment effects on the brain and how these contribute to symptom changes are mitigated. Clearly, though, a more comprehensive future clinical trial with a larger sample size is warranted.

Third, this study was not designed to identify the extent to which the observed pathology or its change by treatment was either associated with DBD diagnoses, prior exposure to maltreatment, or both. DBD diagnosis and prior trauma exposure commonly co-occur (Bernhard et al. 2018), and both trauma exposure and DBD diagnosis have been associated with heightened responsiveness to facial expressions (McCrory et al. 2017)—particularly if DBD is associated with trauma exposure (Meffert et al. 2018). As such, it is possible that the result of this study should only be considered in the context of treating youths with DBD diagnoses and a history of trauma exposure (not youths with DBD without trauma history or youths with trauma exposure and mental health concerns but without DBD diagnosis). However, it has to be noted that neural areas showing significant differences between healthy youths and youths with DBDs are implicated both in the pathophysiology of DBDs and trauma exposure/PTSD (Blair et al. 2013; Andrewes and Jenkins 2019). However, we also acknowledge the need for future work with larger *N*s.

## Conclusions

In summary, we report this preliminary result of the effectiveness of fluoxetine treatment on youths with DBDs and history of trauma exposure. We observed youths with DBDs, and history of trauma showed significant improvement in the core DBD symptoms after fluoxetine treatment. Moreover, we observed increased recruitment of regions implicated in top-down attention control areas and emotional responding/emotion regulation area after fluoxetine treatment. Notably, there were significant associations between improvements in core DBD symptomatology and increased neural recruitment. This may provide guidance to the future direction of the clinical study and treatment for this challenging population.

## Clinical Significance

Our findings have an important implication for the population who struggle with significant mental health issues of DBD symptoms and trauma exposure symptoms. We demonstrated the impact of a serotonergic agent (fluoxetine) on symptom profiles and neural response in youths with DBDs and a history of trauma exposure. Especially after 6 weeks of fluoxetine treatment, youths with diagnoses of DBDs and history of trauma exposure demonstrated significant level of symptom improvement in externalizing problems, aggressive behavior, ODD symptoms, irritability, anxiety-depression symptoms, as well as trauma-related symptoms. Clinically this suggests that youths with DBDs require careful assessment of the presence of past history of trauma exposure, because they may benefit from serotonergic agents than other psychopharmacological options.

Our findings also provide the possibility of using neural response as a biomarker for treatment response in youth with DBDs and may guide future clinical study and pharmacotherapy for this challenging population.

## Supplementary Material

Supplemental data

Supplemental data

Supplemental data

Supplemental data

Supplemental data

Supplemental data

## Supplementary Material

Supplementary Material

Supplementary Section S1

Supplementary Section S2

Supplementary Section S3

Supplementary Section S4

Supplementary Section S5
