# Supplementary material for: Neural Responses to Fluoxetine in Youths with Disruptive Behavior and Trauma Exposure: A Pilot Study
Source: J Child Adolesc Psychopharmacol. 2021 Oct 14;31(8):562–71. doi: 10.1089/cap.2020.0174 (PMC8575058; doi:10.1089/cap.2020.0174)
Supplement: Supplemental data [file Supp_Data.docx]

**Supplemental Material**

**Supplemental Material Section 1: Clinical assessment/characterization of the participants**

Clinical assessment/characterization was done via psychiatric interviews with the participants and their parents by licensed and board-certified child and adolescent psychiatrists who adhered closely to routine, standard psychiatric clinical practice for this population. A doctoral level researcher or a member of the clinical research team obtained written informed consent and assent. In all cases, children and adolescents had the right to decline participation at any time before or during the study. Consent documents were reviewed with parents/legal guardians and written permission was obtained at the initial visit for community participants or at the time of intake to the outpatient clinic for youths with disruptive behavior disorders.

Youths with disruptive behavior disorders and healthy youths received the Schedule to confirm their diagnoses or rule-out of any psychiatric disorders (in healthy youths) using for Affective Disorders and Schizophrenia for School-Age Children-Present and Lifetime Version (K-SADS)^1^. IQ was assessed with the Wechsler Abbreviated Scale of Intelligence (two-subset form).^2^

**Supplemental Material Section 2: Exclusion Criteria**

Exclusion criteria were: 1. Comorbid psychotic, tic, or pervasive developmental disorders, substance use disorders. 2. Major medical illness that prohibits treatment by fluoxetine (e.g., severe liver disease, seizure disorder, metabolic disorder). 3. History of significant worsening of pre-existing psychiatric symptoms after treatment with serotonergic agents (such as Fluoxetine or Sertraline) such as development of suicidal ideation and/or intent, requirement of psychiatric hospitalization, or serious medical compromises (e.g., serotonin syndrome). 4. History of central nervous system (CNS) disease (including history of seizure, epilepsy, CNS tumor, CNS hemorrhage, or serious CNS infection including meningitis or encephalitis). 5. Current use of any psychiatric medications. 6. A positive urine pregnancy test. 7. A positive urine toxicology. 8. Wechsler Abbreviated Scale of Intelligence scores < 70. 8. Metal in body (i.e., hearing aid, cardiac pacemaker, bone plates, etc.), claustrophobia, or any other condition that would preclude functional MRI (fMRI) scanning.

**Supplemental Material Section 3: Psychiatric Treatment/Dosing and maintenance strategy of fluoxetine treatment**

The decision on initiation of Fluoxetine was a collective clinical decision by youths, their parents, and the clinician (Dr. Chung) who provided psychiatric assessment/treatment. Youths with DBDs and a history of trauma exposure and their parents were offered the option of medication treatment with fluoxetine. The clinician explained that the decision-making process regarding this medication option was not a part of the study. They were given the opportunity of participating in the study without fluoxetine treatment as well. However, they were informed that they could continue study participation or withdraw from the study regardless of medication treatment status. All the participants of youths with -DBDs recruited to the study showed significant levels of symptoms and functional impairment eligible for medication treatment, which was determined by the clinical assessment and interview. There was no participant who was previously treated with Fluoxetine or any other serotonergic agent in the past.

If the participant and his/her legal guardian agreed with medication treatment, fluoxetine treatment was started as 5mg/day in the morning. This dose was increased 10mg/day at week 2, and 20mg/day at week 3. The dose titration was determined by the clinician’s judgement. At the end of week 3, if there was an inadequate response but good tolerability of the medication, the dose was increased beyond 20mg until it reached either an optimal dose or there was adverse effect which precluded further increase of the dose. If response is good to excellent and tolerability is at least good at the end of week 3, the dose will be maintained as 20mg until the end of the study participation. At the follow-up visits after week 3, if the initial good to excellent response is not maintained, the dose can be increased afterwards. Likewise, at the end of week 3, if the fluoxetine dose is not adequately tolerated, the dose can either be maintained (allowing additional time to accommodate) or lowered back to 10mg or 5mg by the decision among the clinician, the participant, and his/her legal guardian. If the tolerability improves, the dose may have been increased again. The participants were followed at the outpatient clinic with weekly visits. For non-response or poor tolerability, the visit interval could be reduced to three days, but none of the participants required this. The average Fluoxetine dose at the end of the 6 week was 26mg (SD=8.2)/0.47mg/kg (SD=0.15).

If the participant and his/her parent did not want to receive medication treatment, the participant and his/her parents were still asked if they were willing to continue study participation. If they agreed, the participants and their parents visited the outpatient clinic weekly to assess symptom level, to discuss coping/management skills for psychiatric symptoms, and general guidelines to improve functioning at school and home. These sessions were also provided for the participants who received fluoxetine. If there was significant worsening of symptom profiles during the follow-ups and/or changes of participants/parents’ decision on medication treatment, medication treatment was provided accordingly with clinical judgement of the child and adolescent psychiatrist, and the participant withdrew from the study.

**Supplemental Material Section 4: The fearful facial expression task**

Participants viewed images of 10 men and women from the Pictures of Facial Affect series.^3^ These images displayed expressions ranging between neutral and 150% fearful affect with the fearful expressions showing parametrically modulated intensity (50%, 100%, and 150% intensity). Intensity was modulated by morphing neutral and 100% fearful expressions to create composites (50% intensity) or extrapolating from emotional expressions to create exaggerated expressions (150% intensity). Because neutral expressions may appear threatening,^4^ we followed previous studies in morphing neutral and happy expressions to create 25% happiness expressions, which are seen as affectively neutral.^5^ Expressions were presented in random order across participants. In keeping with the design of previous studies, participants performed an implicit processing task in which they indicated the gender of the faces using two response buttons. This method has been found to enhance blood oxygen level dependent (BOLD) responses to emotional expression stimuli.^6^ Responses and latencies were recorded. Stimuli were presented on a computer display that was projected onto a mirror in the MRI scanner. Stimulus presentation occurred in one run, which lasted 5 minutes and 50 seconds, and compromising 160 randomly ordered events (80 face trials (20 trials for each intensity) consisting of a 2-second face presentation and a 900-millisecond fixation cross, and 80 interspersed “jittered” trials). Runs were preceded by four fixation trials and concluded by five fixation trials (each 3 seconds in length).

**Supplemental Material Section 5. Image Data Preprocessing**

The fMRI data were preprocessed and analyzed with the Analysis of Functional NeuroImages (AFNI) software package.^7^ Both individual and group level analyses were conducted. At the individual level, functional images from the first four repetitions collected prior to equilibrium magnetization were discarded, leaving 680 repetition times per participant. The participants’ anatomical scans were then individually registered to the Talairach and Tournoux atlas.^8^ The individuals’ functional EPI data were then registered to their Talairach anatomical scan. The EPI datasets for each participant were spatially smoothed (isotropic 6 mm^3^ Gaussian kernel) to reduce variability among individuals and generate group maps. Next, the time series data were normalized by dividing the signal intensity of a voxel at each time point by the mean signal intensity of that voxel for each run and multiplying the result by 100, producing regression coefficients representing percent-signal change.

Excessive movement was defined as occurring if a participant had >10% of TRs discarded due to movement above study limits (1.0cm). Four youths with disruptive behavior disorders and one healthy youth were not included in the final analyses due to excessive movement. We conducted three group (healthy youths, youths with DBDs who received fluoxetine treatment, youths with DBDs who did not receive fluoxetine treatment) ANOVAs on the motion regressors. There were no significant group differences in movement parameters, for the youths included in the final group analyses; [F(1,38)=1.351-2,439, p>0.1].

At the individual level processing, our goal was to identify neural areas showing differential signal strength according to the intensity of emotional expression. As such, a model was generated using six motion regressors and the flowing three regressors: fearful facial expression, fearful facial expression weighted according to emotion intensity, and incorrect responses. GLM fitting was performed with these three regressors, six motion regressors and a regressor modeling a first-order baseline drift function. All regressors were convolved with a canonical hemodynamic response function (HRF) to account for the slow hemodynamic response (with time point commencing at time of first image onset). This produced a β coefficient and associated *t* statistic for each voxel and regressor. There was no significant regressor collinearity.

**Supplemental Material Section 6: T-test between healthy youths and youths with DBDs**

To assess group differences in baseline DBD symptom levels (externalizing problems, breach of rules, aggression, Oppositional Defiant Disorder symptoms, Conduct Disorder symptoms, and irritability), CBCL-indexed depressive-anxiety symptoms, and trauma-related symptoms (i.e., CROPS and PROPS), we performed eight 3 (Group) one-way ANCOVAs on the DBD symptoms, anxiety-depressive symptoms of CBCL/trauma-related symptoms of CROPS/PROPS with age, gender, and IQ as covariates. Bonferroni correction was applied for the threshold of statistical significance [p<0.05/9=0.0056].

We also contrasted the modulated BOLD responses of the youths with DBD and the typically developing youths at baseline to fearful expressions. Areas showed significant group differences included left ventromedial prefrontal cortex, superior parietal lobule, lingual gyrus and amygdala; see Table S3. For left superior parietal lobule, lingual gyrus, and ventromedial prefrontal cortex, typically developing youths showed greater modulated BOLD responses to fearful expression relative to youths with DBDs. In left amygdala, youths with DBDs showed greater modulated BOLD responses to fearful expression relative to healthy youths.

**Supplemental Material Section 7: Main effect of group and time**

Areas showing a main effect of group included left superior frontal gyrus, left precuneus, right post-central gyrus, right paracentral lobule, left superior parietal lobule, left insula, left parahippocampal gyrus, and left ventromedial prefrontal cortex, see Table S4. In all cases, the weighted BOLD responses to fearful expression were significantly increased in healthy youths compared to youths with DBDs.

Areas showing a main effect of time included left precentral gyrus, left postcentral gyrus, and left amygdala; see Supplemental Material Table S4. For left precentral gyrus and postcentral gyrus, the weighted BOLD responses to fearful expressions were significantly increased after treatment, relative to baseline. For left amygdala, the weighted BOLD responses to fearful expression were significantly decreased after treatment relative to baseline. Given the interest regarding potential treatment effect by Fluoxetine on this area, we have conducted an ad-hoc analysis on the BOLD responses in this area to the weighted fearful facial expression. This showed that indeed youths with DBDs and Fluoxetine treatment showed more significant decrease of the activation of this area after Fluoxetine treatment, compared to youths with DBDs and without Fluoxetine treatment, and healthy youths [F=4.712, η ^2^ =0.112, p=0.012]; see Figure S1.
