# Supplementary material for: Neural Responses to Fluoxetine in Youths with Disruptive Behavior and Trauma Exposure: A Pilot Study
Source: J Child Adolesc Psychopharmacol. 2021 Oct 14;31(8):562–71. doi: 10.1089/cap.2020.0174 (PMC8575058; doi:10.1089/cap.2020.0174)
Supplement: Supplemental data [file Supp_TableS1.docx]

Table S1. Symptom profiles in baseline.

|  | Healthy youths  (n=18) | Youths with DBDs on Fluoxetine treatment (n=11) | Youths with DBDs without Fluoxetine treatment (n=10) | P value  (DBD vs Healthy) |
| --- | --- | --- | --- | --- |
|  | Mean (SD) | | | |
| Externalizing Problems | 51.17 (9.12) | 83.45 (7.15) | 78.30 (3.62) | <0.001 |
| Breach of Rules | 53.61 (5.04) | 75.64 (6.96) | 71.90 (3.78) | <0.001 |
| Aggressive Behavior | 53.72 (4.92) | 73.27 (7.51) | 71.40 (11.33) | <0.001 |
| Oppositional Defiant Disorder Symptoms | 53.50 (4.99) | 76.27 (9.81) | 77.40 (9.13) | <0.001 |
| Conduct Disorder Symptoms | 53.11 (5.40) | 75.00 (5.46) | 71.60 (3.57) | <0.001 |
| Irritability | 0.89 (0.90) | 5.45 (0.82) | 5.50 (0.53) | <0.001 |
| Anxiety-Depression | 52.33 (4.28) | 65.55 (9.64) | 60.60 (7.14) | <0.001 |
| CROPS | 8.22 (8.27) | 19.18 (7.57) | 17.30 (7.51) | <0.001 |
| PROPS | 5.00 (3.74) | 16.55 (12.45) | 14.10 (7.89) | <0.001 |

Abbreviations: DBDs, Disruptive Behavior Disorders; SD, Standard Deviation; CROPS, Child Report of Posttraumatic Symptoms; PROPS, Parent Report of Posttraumatic Symptoms
