# Supplementary material for: Neural Responses to Fluoxetine in Youths with Disruptive Behavior and Trauma Exposure: A Pilot Study
Source: J Child Adolesc Psychopharmacol. 2021 Oct 14;31(8):562–71. doi: 10.1089/cap.2020.0174 (PMC8575058; doi:10.1089/cap.2020.0174)
Supplement: Supplemental data [file Supp_TableS2.docx]

Table S2. Symptom profiles in follow-up.

|  | Healthy youths  (n=18) | Youths with DBDs on Fluoxetine treatment (n=11) | Youths with DBDs without Fluoxetine treatment (n=10) | P value  (Difference between baseline and follow-up) |
| --- | --- | --- | --- | --- |
|  | Mean (SD) | | | |
| Externalizing Problems | 51.44 (7.96) | 66.27 (5.95) | 76.80 (3.85) | <0.001** |
| Breach of Rules | 53.78 (5.55) | 74.09 (6.82) | 71.80 (3.77) | 0.497 |
| Aggressive Behavior | 54.17 (4.27) | 64.64 (5.99) | 72.90 (8.44) | <0.001** |
| Oppositional Defiant Disorder Symptoms | 54.06 (4.92) | 64.55 (10.08) | 76.50 (7.71) | <0.001** |
| Conduct Disorder Symptoms | 53.22 (5.67) | 72.64 (7.42) | 69.80 (5.33) | 0.240 |
| Irritability | 0.94 (0.94) | 3.09 (0.94) | 5.60 (1.51) | <0.001** |
| Anxiety-Depression | 52.61 (4.22) | 56.91 (6.83) | 62.80 (4.94) | <0.001** |
| CROPS | 9.06 (8.08) | 15.00 (4.82) | 18.70 (6.62) | 0.017* |
| PROPS | 5.39 (5.75) | 11.82 (5.71) | 16.70 (7.41) | 0.043* |

*p<0.05, **p<0.005

Abbreviations: DBDs, Disruptive Behavior Disorders; SD, Standard Deviation; CROPS, Child Report of Posttraumatic Symptoms; PROPS, Parent Report of Posttraumatic Symptoms
