# Supplementary material for: Neural Responses to Fluoxetine in Youths with Disruptive Behavior and Trauma Exposure: A Pilot Study
Source: J Child Adolesc Psychopharmacol. 2021 Oct 14;31(8):562–71. doi: 10.1089/cap.2020.0174 (PMC8575058; doi:10.1089/cap.2020.0174)
Supplement: Supplemental data [file Supp_FigS1.docx]

Figure S1. Symptom profile changes between pre-treatment (1) and post-treatment (2) among youths with DBDs and history of trauma exposure who received Fluoxetine treatment (blue -), youths with DBDs and history of trauma exposure who did not receive Fluoxetine treatment (red -), and health youths (green -); (A) CBCL externalizing problems; (B) CBCL aggressive behavior; (C) CBCL Oppositional Defiant Disorder symptoms; (D) CBCL irritability; (E) CBCL anxiety-depression; (F) CROPS; (G) PROPS; (H) CBCL breath of rules; (I) CBCL Conduct Disorder symptoms. (A)-(G): significant group by time interaction [p<0.001]. (H)-(I): no significant group by time interaction [p=0.240-0.497].


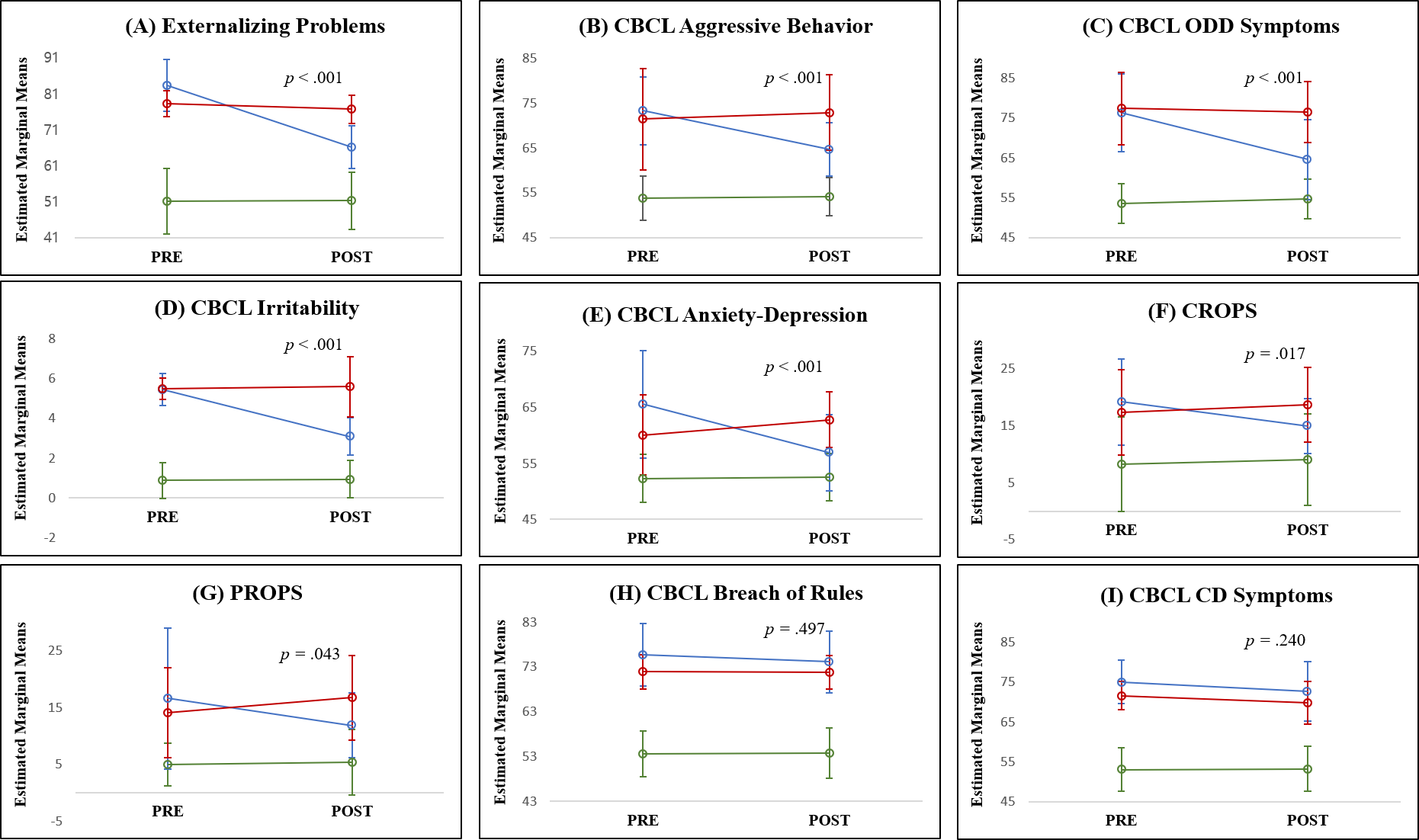


Abbreviations: DBDs, Disruptive Behavior Disorders; CBCL Child Behavior Checklist; CROPS, Child Report of Posttraumatic Symptoms; PROPS, Parent Report of Posttraumatic Symptoms.
