# Supplementary material for: Neural Responses to Fluoxetine in Youths with Disruptive Behavior and Trauma Exposure: A Pilot Study
Source: J Child Adolesc Psychopharmacol. 2021 Oct 14;31(8):562–71. doi: 10.1089/cap.2020.0174 (PMC8575058; doi:10.1089/cap.2020.0174)
Supplement: Supplemental data [file Supp_TableS4.docx]

Table S4. Brain regions showing significant main effects of group and time.

|  | Coordinates of peak activation^b^ | | | | |  |  |  |
| --- | --- | --- | --- | --- | --- | --- | --- | --- |
| Region^a^ | Left/Right | BA | x | y | z | F | Voxels | η^2^ |
| **Main effect of Group** |  |  |  |  |  |  |  |  |
| Superior frontal gyrus | Left | 8 | -10 | 31 | 44 | 13.17 | 38 | 0.112 |
| Precunues | Left | 7 | -4 | -40 | 47 | 10.19 | 98 | 0.137 |
| Postcentral gyrus | Right | 2 | 52 | -19 | 32 | 10.68 | 56 | 0.115 |
| Paracentral lobule | Right | 5 | 19 | -43 | 53 | 11.79 | 75 | 0.127 |
| Superior parietal lobule | Left | 5 | -19 | -40 | 59 | 9.50 | 46 | 0.121 |
| Insula cortex | Left | 13 | -34 | -19 | 14 | 8.11 | 35 | 0.109 |
| Parahippocampal gyrus | Left | 36 | -34 | 34 | -9 | 13.04 | 75 | 0.126 |
| Ventromedial prefrontal cortex | Left | 10 | -4 | 49 | -3 | 12.00 | 31 | 0.100 |
| **Main effect of Time** |  |  |  |  |  |  |  |  |
| Precentral gyrus | Left | 4 | -46 | -13 | 38 | 18.31 | 60 | 0.130 |
| Postcentral gyrus | Left | 3 | -55 | -13 | 23 | 14.23 | 44 | 0.129 |
| Amygdala | Left |  | -16 | 1 | -18 | 20.81 | 28 | 0.105 |

^a^According to the Talairach Daemon Atlas (<http://www.nitc.org/projects/tal-daemon/>).

^b^Based on the Tournoux and Talairach standard brain template.
