# Supplementary material for: Neural Responses to Fluoxetine in Youths with Disruptive Behavior and Trauma Exposure: A Pilot Study
Source: J Child Adolesc Psychopharmacol. 2021 Oct 14;31(8):562–71. doi: 10.1089/cap.2020.0174 (PMC8575058; doi:10.1089/cap.2020.0174)
Supplement: Supplemental data [file Supp_TableS3.docx]

Table S3. Brain regions showing significant difference between healthy youths and youths with DBDs in baseline.

|  | Coordinates of peak activation^b^ | | | | |  |  |  |
| --- | --- | --- | --- | --- | --- | --- | --- | --- |
| Region^a^ | Left/Right | BA | x | y | z | F | Voxels | η^2^ |
| **Healthy Youths > Youths with DBDs** |  |  |  |  |  |  |  |  |
| Superior parietal lobule | Left | 7 | -19 | -40 | 47 | 4.23 | 244 | 0.214 |
| Lingual gyrus | Left | 19 | -7 | -64 | 2 | 4.02 | 24 | 0.101 |
| Ventro-medial prefrontal cortex | Left | 32 | -4 | 40 | -3 | 5.17 | 24 | 0.102 |
| **Healthy Youths < Youths with DBDs** |  |  |  |  |  |  |  |  |
| Amygdala | Left |  | -25 | -1 | -21 | 3.69 | 46 | 0.161 |

^a^According to the Talairach Daemon Atlas (<http://www.nitc.org/projects/tal-daemon/>).

^b^Based on the Tournoux and Talairach standard brain template.
